# Gesture sonification for enhancing agency: an exploratory study on healthy participants

**DOI:** 10.3389/fpsyg.2024.1450365

**Published:** 2025-02-10

**Authors:** Felix Schoeller, Parham Ashur, Joseph Larralde, Clement Le Couedic, Rajeev Mylapalli, Karthikeyan Krishnanandan, Anna Ciaunica, Adam Linson, Mark Miller, Nicco Reggente, Vladimir Adrien

**Affiliations:** ^1^Institute for Advanced Consciousness Studies, Santa Monica, CA, United States; ^2^Media Lab, Massachusetts Institute of Technology, Cambridge, MA, United States; ^3^Emlyon Business School, Ecully, France; ^4^Independent Developer, Paris, France; ^5^Aura Healthcare, Paris, France; ^6^Centre for Research and Interdisciplinarity, University of Paris, Paris, France; ^7^Centre for Philosophy of Science, Faculty of Science, University of Lisbon, Lisbon, Portugal; ^8^Institute of Cognitive Neuroscience, University College London, London, United Kingdom; ^9^School of Computing and Communications, Open University, Edinburgh, United Kingdom; ^10^Center for Human Nature, Artificial Intelligence and Neuroscience, Hokkaido University, Sapporo, Japan; ^11^AP-HP, Department of Psychiatry, Avicenne Hospital, Paris Nord Sorbonne Université, Bobigny, France; ^12^Université Paris Cité, Inserm, UMR-S 1266, Institute of Psychiatry and Neuroscience of Paris, INSERM U1266, Paris, France

**Keywords:** proprioception, agency, body awareness, body ownership, dance movement therapy, mental health, sensorimotor, cognitive augmentation

## Abstract

**Background:**

Body awareness (BA) and proprioception, which are essential components of the sense of agency (SA), are often altered in various mental disorders such as posttraumatic stress disorder (PTSD). However, the relationship between BA, proprioception, and SA, as well as the methods to manipulate them, remain unclear. This study explored using real-time gesture sonification (GS), i.e., wearable technology transforming body movements into sounds, to enhance proprioception, BA, and thus the SA.

**Methods:**

In this within-subjects design, 17 healthy adults (mean age = 25.5 years) with varying dance expertise (novice, amateur, expert) improvised movements to match sounds with and without auditory feedback from motion sensors on wrists/ankles modulated by their gestures. BA, immersion, pleasure, and self-efficacy were measured.

**Results:**

Sonification significantly increased body awareness, reward, and immersion (all *p* < 0.05).

**Conclusion:**

GS can enhance BA and the SA, pleasure, and control during physical activity. This highlights potential mental health applications, such as agency-based therapies for PTSD. Manipulating bodily perception could improve symptoms and embodiment. Further research should replicate this in clinical populations and explore neurocognitive mechanisms.

## 1 Introduction

Previous work has highlighted the benefits of dance and physical activity on human well-being, mental health, and neurorehabilitation (Bar et al., 2021; Mikkelsen et al., 2017; Bearss et al., 2017). For example, dancing has been effective in mitigating symptoms of post-traumatic stress disorder, depression, and anxiety (Marchant et al., 2010; Aguiar et al., 2016; Westheimer et al., 2015). However, the relationship between proprioception—the perception of the body in space and a key component of body movements and exercise (Barlow, 2017)—and body awareness (BA) is still unclear (Mehling, 2020; Bernard and Mittal, 2015). BA is typically defined as the ability to perceive and interpret internal state of one’s body, integrating signals from interoceptive (temperature, pain, cardiac signals, and respiration), proprioceptive and external streams (Gallagher, 2000). Everyday experience also involves the sense of agency (SA); namely, the feeling that ‘I am in control of my own actions, and leverage them to access or change the external world’ (Gallagher, 2000; Haggard, 2017). The integration of sensory information across multiple channels is fundamental to building a cohesive representation of the environment and of our body (Pezzulo, 2014). Such continuous updating of the body’s representation gives rise to the formation of a unitary body image and the subjective experience of being present in the here and now (Varela et al., 2017).

While the role of interoceptive channels (cardioception, respiration, gastric, temperature, etc.; Craig, 2002) has long been a topic of research in mental health through, e.g., biofeedback and meditation (Schoenberg and David, 2014; Weerdmeester et al., 2020; Schoeller et al., 2024), the importance of motor processing in psychopathology has been largely understudied (Bernard and Mittal, 2015; Garvey and Cuthbert, 2017). Indeed, some of the most striking symptoms in psychopathology often include the motorium [e.g., functional neurological disorder, catatonia, hypo-kinesia, or freezing states in posttraumatic stress disorder (PTSD; Adrien et al., 2024)]. Proprioception—the perception of the body in space—has been highlighted as a key dimension for diagnosing, monitoring and treating mental illnesses (Bernard and Mittal, 2015). However, at present, research on the role of proprioception in well-being and mental health (e.g., aging effects, injury rehabilitation, living with Parkinson’s) largely centers on body coordination, fluidity of movement, posture, balance, and stability (Marchant et al., 2010; Aguiar et al., 2016; Westheimer et al., 2015). Far less is known about how proprioception relates to experiential judgments that may play a role in emotional regulation, beyond movement itself (Mehling, 2020). For instance, the enactive perspective of PTSD sees psychological trauma as a breakdown of SA explaining its symptoms as an adaptive response (Adrien et al., 2024). Crucially, there are limited methods to intervene on and manipulate BA or the SA in people during physical activities (see 21, 22, 9). However, the flexible nature of BA offers potential for manipulating our SA. This malleability is strikingly illustrated by the rubber hand illusion (RHI), where multisensory manipulation alters the sense of body ownership (SO, a significant component of BA) even without direct tactile stimulation (Samad et al., 2015). The proprioceptive drift experienced by participants is higher for patients with the dissociative subtype of PTSD (Rabellino et al., 2018) showing that their SO is disminished. The RHI has been adapted to a *dynamic* RHI (Kalckert and Ehrsson, 2012) that can also manipulate the SA.

Harnessing this underutilized potential for manipulation, the current study leveraged gesture sonification (GS), a system that involves using body gestures to control and generate sounds, augmenting movement with real-time sound effects in order to manipulate BA and SA during physical exercise. It uses sensors to detect the movement of an individual’s body and translates this movement into sound. This study extends mental health research on GS (Vidyarthi and Riecke, 2014) to the proprioceptive modality. The dynamic nature of mental body representation, continuously updated through sensory feedback including sound (Haar et al., 2020), suggests the potential for novel therapeutic sensory approaches in addressing BA alterations (Tajadura-Jiménez et al., 2017; Schoeller et al., 2019). The field of interoceptive engineering and body illusions has emerged as a means to study and train body perception (Schoeller et al., 2019; Matamala-Gomez, 2020; Bevilacqua et al., 2016). For example, Iodice et al. have shown that (false) auditory feedback of heart-rate can influence the way we evaluate our (cardiac) interoceptive state, and thereby affect the perception of effort level during physical exercise (Iodice et al., 2019). GS is used in the context of multimodal sensory integration to enhance motor learning (Sigrist et al., 2013; Lauber and Keller, 2014). It has also been used as a stroke rehabilitation strategy and in sports (Fritz et al., 2013), where it was found to have potential for diminishing pain or effort perception due to an increased SA. This exploratory study investigated the use of GS to manipulate BA in healthy populations, comparing responses across dance expertise levels (novice, amateur, expert). We hypothesized that participants using GS would improve their BA, and—as they could better perceive the (immediate) sensory consequences of their physical gestures—this should enhance their SA and the pleasure resulting from this increased sensation of control.

## 2 Methods

### 2.1 Procedure

**Figure 1:**
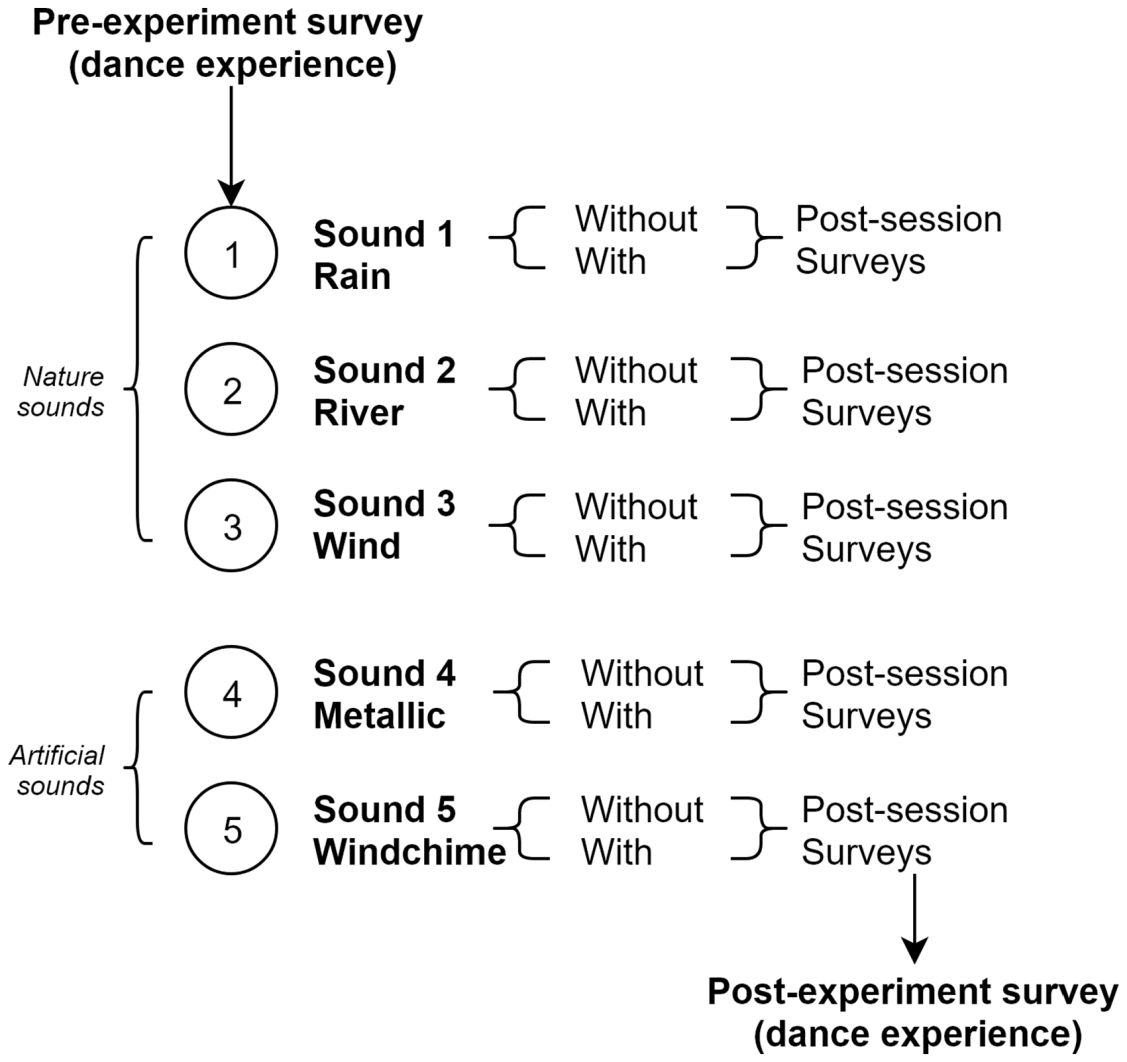
Experimental timeline (within-subject design). Participants are exposed to sounds with and without the device, producing corresponding behaviors.

The accelerometer sensors were attached to participants’ wrists and ankles in the laboratory. During each trial, participants were first exposed to a sound and asked to create a movement that satisfyingly embodies the sound. The experimenter measured how long it took them to produce the movement until they were satisfied, then participants answered a series of questions about this experience. In the second phase, the GS device was turned on and the procedure was repeated, except this time participants wore the GS device and their actions were modulating the sound output (i.e., they had proprioceptive control). The session was divided into 5 trials (type of sounds) × 2 (with/without proprioceptive control) sections each (Figure 1). After the 5 sounds series, participants answered a questionnaire about the experience and the experimenter fully debriefed them. Each participant was happy about the experiment and reportedly did not see the time pass. They were thrilled about the potential of the technology and eager to find out how it could be used in everyday life. Each experiment lasted approximately 30 min.

### 2.2 Participants

The study included 17 participants (12 women; M_age_ = 25.5 years; M_height_ = 168.4 cm). They were divided into dance experience groups: professional dancers practicing everyday for more than 10 years (n = 5; 4 women; M_age_ = 27 years; M_height_ = 166.8 cm), amateur dancers practicing once a week as a hobby since 1–10 years (n = 6; 4 women; M_age_ = 25.5 age; M_height_ = 169.1 cm), and novices who do not practice regularly, only dance on very rare occasions or do not dance at all (*n* = 6; 4 women; M_age_ = 23.8 years; M_height_ = 169 cm). Participants were randomly assigned to one of two experimental conditions. In a counterbalanced within-subject design, each participant experienced both conditions: with the device on and with the device off.

### 2.3 Materials

#### 2.3.1 Wearables

##### 2.3.1.1 System architecture

**Figure 2:**
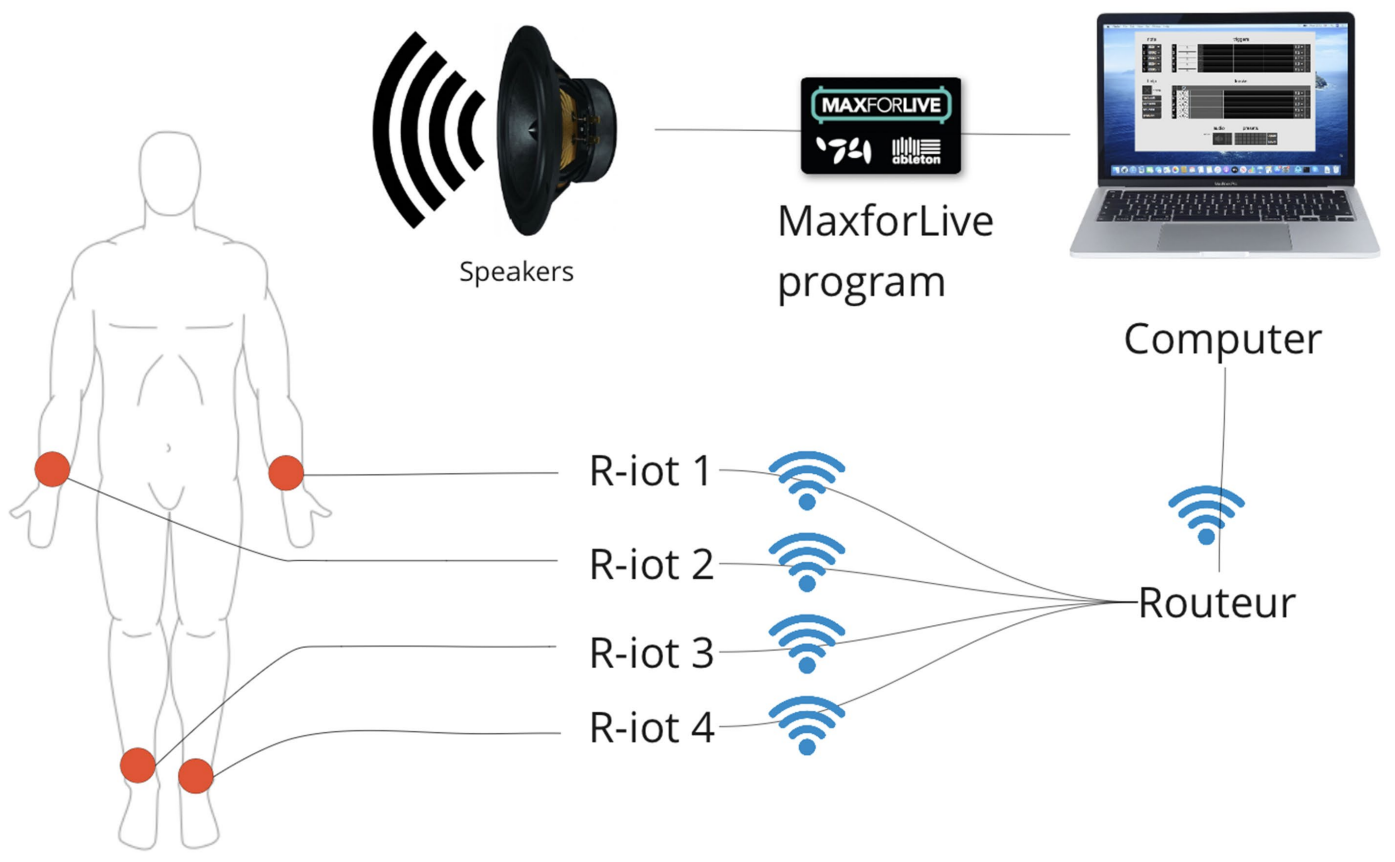
System architecture depicting four sensors (right and left wrists and ankles) capturing participant movement intensity. BITalino R-IoT is a device that integrates a 9-axis sensor and onboard computation to determine its absolute orientation in space. Data is transmitted via WiFi to a Max program, where it is used to modulate the volume of the sound output.

Each device is attached to participants’ wrists and ankles. The sensors’ raw movement data is streamed to software to transform the energy of the movement into sound and broadcast the audio output live through speakers (Figure 2).

##### 2.3.1.2 Sensors

The sensor used was a 34 × 23 × 7 mm R-IoT by Bitalino including a Triaxial Accelerometer (ACC), Triaxial Gyroscope (GYR) and Triaxial Magnetometer (MAG), and communicating via 2.4GHz WiFi to the standalone software. It is powered by a 500 mAh Li-Po Battery. The sensor is wrapped into a 3D printed case and into a comfortable cotton bracelet to attach it to the wrist and ankles of the dancers.

##### 2.3.1.3 Software

We developed a custom Max patch for real-time soundtrack generation during movement performance. The patch accommodates up to five WiFi-connected R-IoT devices, each assignable to an individual audio track. The program is a Max patch designed and developed for the project. It allows users to connect up to 5 R-IoT devices via WiFi and use them to generate a real-time soundtrack of the performance/ Each connected R-IoT can be assigned to a track. An accelerometer-derived intensity value, rather than a physically rigorous energy measurement, was used to modulate the volume. The value used to modulate the volume is obtained by processing the accelerometer data. It is not a strict energy value in the scientific sense, which is why it is rather named “intensity.” The three accelerometer axes were received in G unit. First, a derivative filter (a linear regression computed on the last three values) is applied to each axis. The absolute value of each derivative is then fed into a weighted integrator, which is equivalent to an IIR filter. This one-dimensional output is then processed by two operators, providing the user with two comprehensive sensitivity parameters: gain and smooth, each controllable by a knob on the user interface. The gain operator is a pow function followed by a multiplication, and the smooth operator is a ramping function with different ramp durations depending on whether the value is going up or down. Each knob has a normalized value mapped empirically to the internal parameters of the operators. All the software described in this article is available upon demand to corresponding authors. The main file to open with Max to run the program is kampnagel.maxpat. More documentation on how to use the patch is included inside and accessible from the main interface.

#### 2.3.2 Sound

Audio output: For the audio output, we used Philips SPA5300 multimedia speakers 2.1 with a 100 W subwoofer at a constant volume throughout the experiments and across the participants.

Sound library: Using the website “freesound.org,” we built a sound library tailored to the experiment including the sounds of fire, water, wind, metallic, and wind chimes.

### 2.4 Ethics statement

The experiment is in compliance with the Helsinki Declaration. All participants gave their voluntary informed consent and we followed the Ethics Code of the American Psychological Association. An independent panel of researchers at the Centre for Research and Interdisciplinarity in Paris, France approved of this protocol and authorized this study in the context of workshops on citizen science experimentation. All participants were informed about the purpose of the research, about their right to decline to participate and to withdraw from the experiment and about the limits of confidentiality. We also provided participants with a contact for any questions concerning the research and with the opportunity to ask any questions regarding the phenomenon under study and receive appropriate answers. All participants reacted positively to the experiment and were thankful for the opportunity.

### 2.5 Reviewer disclosure

Following the standard reviewer disclosure request endorsed by the Center for Open Science (Nosek et al., 2017; Bafeta et al., 2020), we confirm to have reported all measures, conditions, data exclusions and how we determined our sample sizes.

## 3 Results

**Figure 3:**
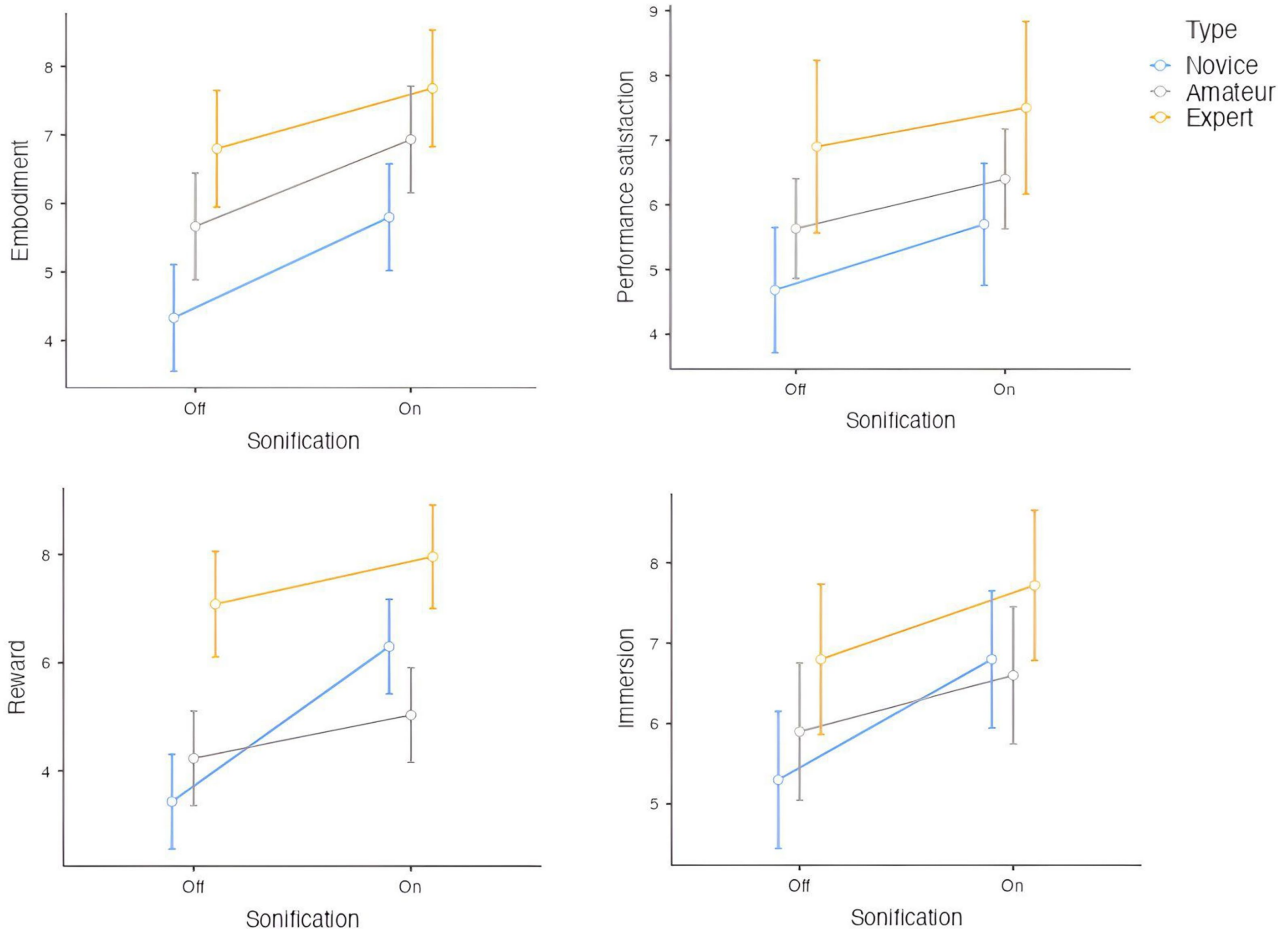
Comparison of embodiment, performance satisfaction, reward, and immersion across different expertise levels (Novice, Amateur, and Expert) and sonification conditions (Device Off vs. Device On). Across all measures, experts consistently reported higher scores compared to novices and amateurs. For all participants, turning the sonification device on increased embodiment, performance satisfaction, reward, and immersion. The largest improvements in scores with the device on are observed for novices, suggesting that sonification may have a greater impact on individuals with less experience. Error bars represent standard deviations.

The 17 participants in the experiment self-reported their level of dance experience (novice, amateur, expert). To test for a main effect of GS on the variables of interest, we first examined whether the distribution of differences between conditions (with and without GS) was normal. We performed a Shapiro–Wilk normality test and found that the distribution of the differences in pairs is significantly different from the normal distribution for embodiment (W = 0.83797, *p*-value <0.0001), performance satisfaction, and self-efficacy (W = 0.73525, *p*-value <0.0001), dance pleasure (W = 0.84301, *p*-value <0.0001) and immersion (W = 0.84072, *p*-value <0.0001; Figure 3).

**Figure 4:**
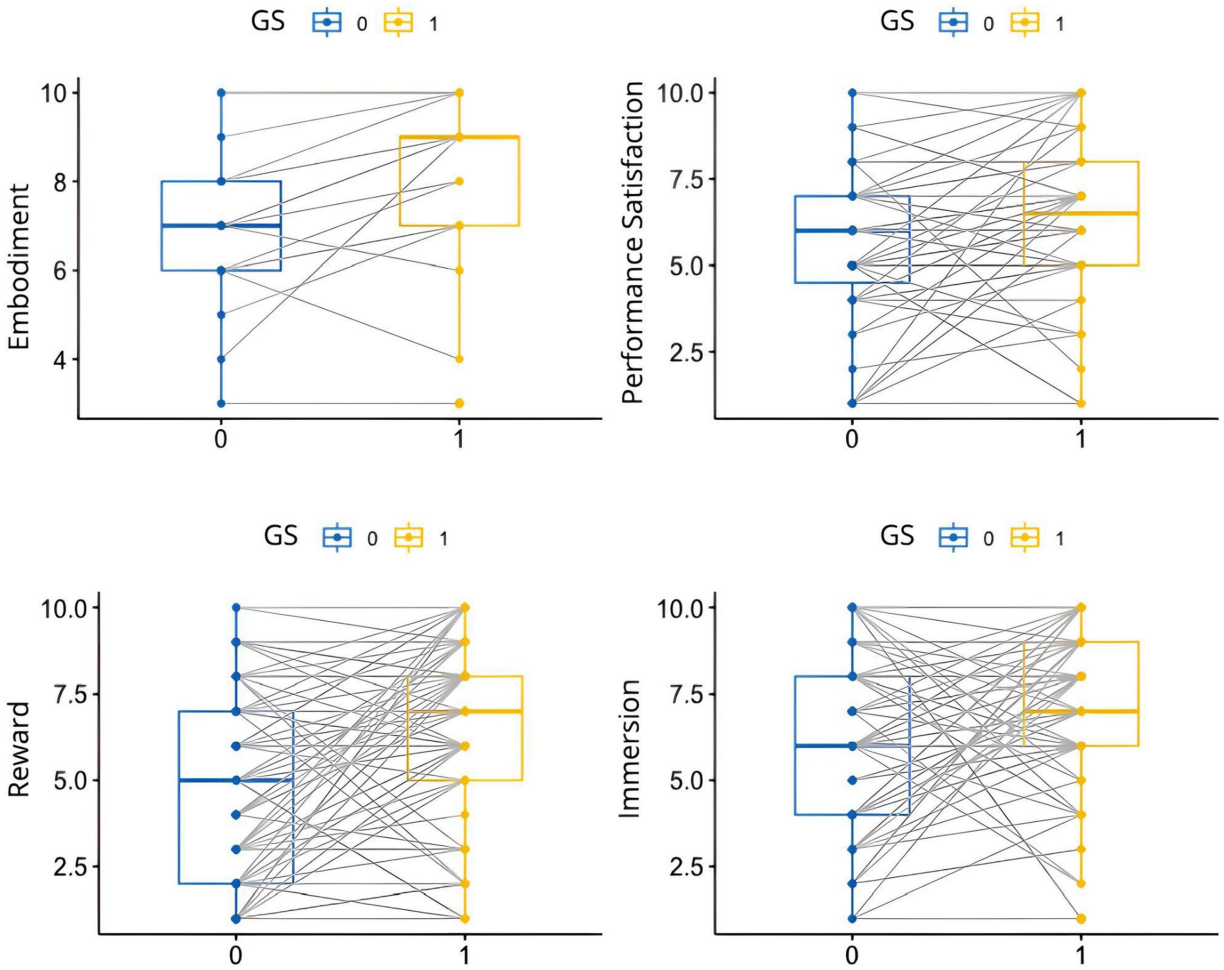
Boxplot comparisons with and without the gesture sonification (GS) device for variables of interest. Each line connects the same participant’s scores across the two conditions. This allows us to see how each participant’s scores change between conditions.

As a result, to examine the effects of GS on the performance, we use the Wilcoxon signed-rank test, a non-parametric test. We find that GS significantly increases embodiment (V = 1395.5, *p*-value <0.0001), immersion (V = 1285.5, *p*-value <0.0001), reward (V = 1,018, *p*-value <0.0001), and performance satisfaction (V = 595.5, *p*-value <0.0001). A Wilcoxon signed rank test with continuity correction showed that GS also significantly increases BA during the performance (V = 90.5, *p*-value = 0.01601). Figure 4 shows the comparisons of the two groups, with or without GS.

**Figure 5:**
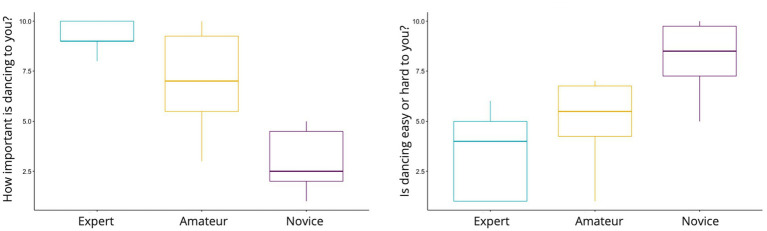
Distribution of self-reported dance difficulty and importance by expertise level. Responses suggest a trend where greater expertise aligns with decreased difficulty and increased personal significance.

We then examined the difference across groups (Novice, Amateur, and Expert). A Kruskal-Wallis test was conducted to compare the effects of Group on Reward, Immersion, Performance Satisfaction, and Embodiment (Figure 5). The results showed a significant effect of Group on Reward, *χ*^2^(2) = 32.87, *p* < 0.001, ε^2^ = 0.1957, and follow-up pairwise comparisons using Dwass-Steel-Critchlow-Fligner tests indicated that Experts had significantly higher rewards compared to both Novices (*p* < 0.001) and Amateurs (*p* < 0.001), while no significant difference was found between Novices and Amateurs (*p* = 0.958). For Performance Satisfaction, there was a significant effect of Group, *χ*^2^(2) = 8.70, *p* = 0.013, ε^2^ = 0.0737. Pairwise comparisons showed that Experts had significantly higher performance satisfaction than Novices (*p* = 0.012), but no significant differences were found between other groups. Lastly, for Embodiment, there was a significant effect of Group, *χ*^2^(2) = 23.68, *p* < 0.001, ε^2^ = 0.1401. Pairwise comparisons revealed that Experts experienced significantly higher embodiment than both Novices (*p* < 0.001) and Amateurs (*p* = 0.011), with no significant difference between Amateurs and Experts (*p* = 0.173). For Immersion, the Kruskal-Wallis test approached significance, *χ*^2^(2) = 5.72, *p* = 0.057, ε^2^ = 0.0339, but pairwise comparisons did not reveal significant differences between any groups.

## 4 Discussion

We examined the effect of proprioceptive and auditory sensory feedback through GS on one’s BA and SA in human healthy adults. By providing dancing participants with GS, we found that they became more aware of their movements with feedback than without. This tentatively confirms the hypothesis that proprioceptive feedback could be used to intervene on BA during physical activity. We also found that participants experienced more pleasure with GS and found the experience more immersive than mere dancing due to increased self-efficacy. These results tentatively confirm our hypothesis that sensory feedback on action and proprioception makes actions more rewarding, likely as a consequence of an increased sense of control and SA. These results confirm that it is possible to use the system described here to manipulate and intervene on BA and SA during proprioceptive exercise. This opens up novel areas of study concerning the relationship between exercise and mental health to test empirically assumptions of existing theories of the role of BA and SA in mental illnesses.

Although experts consistently rated higher in overall embodiment, performance satisfaction, reward, and immersion, the relative change due to GS was more pronounced in less experienced individuals, namely novices and amateurs. This suggests that GS may be especially beneficial for those who have not yet developed heightened proprioceptive awareness. For these individuals, the added sensory feedback may serve as a powerful tool for increasing their sense of agency and bodily control during movement. Interestingly, while immersion did not show significant group differences, the other outcomes highlight the potential for GS to provide additional support for individuals with less expertise, aligning with theories of active inference, where enhanced sensory feedback improves body awareness and self-agency. Future studies should explore whether these effects translate to therapeutic contexts, where increasing bodily awareness can play a crucial role in mental health interventions, particularly for conditions involving disrupted proprioception or agency.

Within the framework of active inference (Friston et al., 2016; Paulus et al., 2019), the main function of perceptual experiences is to fulfill the physiological needs and integrity of the organism (e.g., the human body), within the required limits for survival and reproduction purposes (Seth and Tsakiris, 2018). Incoming sensory inputs are contrasted or ‘matched’ against learned (or innate) patterns constituting what are called ‘predictions’. When a prediction does not match ongoing sensory input, then a ‘prediction error’ (PE) results, which may have the effect of updating the prediction. It has been argued that the basic experience of being a self is the result of an ongoing inferential process based on a generative model centered on the self (Limanowski and Blankenburg, 2013; Apps and Tsakiris, 2014). In order to successfully minimize present and future prediction errors through actions, a person must be able to determine their own uncertainty about its possible actions. This occurs through so-called precision-weighting—where precision weighting refers to the uncertainty or confidence associated with the predictions of an action policy (technically, the inverse variance in the mean of prediction error).

GS theoretically manipulates sensory precision weighting, compelling participants to focus on the sonic consequences of their movement, mandating attention toward their own body. The auditory feedback on the participant’s action provides an exteroceptive scaffolding for proprioceptive signals enhancing motor perception and control. Physiology typically distinguishes between efference (top-down signals from the central nervous system to the periphery—e.g., motor command), and afference (bottom-up sensory information coming from receptors at the periphery—e.g., sound) and their respective neural basis (efferent and afferent nerves). When an efferent signal is produced, it has been suggested that a copy of the signal, known as *an efference copy* or corollary discharge (Latash, 2021), is created so that exafference (sensory signals generated from external stimuli in the environment) can be distinguished from reafference (sensory signals resulting from an animal’s own actions). Hence, the enhancement of reafferent signals with auditory cues reinforces the expectations of participants about their SA. As a consequence, the participant’s feelings of immersion and embodiment are enhanced, while at the same time BA increases since the sound signal carries information about the states of the body in space. We suspect that this effect is strengthened here by the sensorimotor coherence of the real-time sensory feedback and its cognitive correspondence—the match between the content of the sound (e.g., water) and the participant’s intentions and self-representation (e.g., “I am water”).

This hints towards the potential of the technology presented here for mental health. In recent years, there has been increasing interest in the bodily roots of mental illness. While psychiatric symptoms have been reinterpreted in the light of dysfunctional BA (Paulus et al., 2019; Khalsa et al., 2018), therapeutic development (e.g., mindfulness-based therapy) have highlighted the important role of BA training in improving mental health. Crucially, psychomotor dysfunction, as well as loss of SA, lie at the core of multiple psychopathologies such as (among others) schizophrenia (Arnfred et al., 2015), autism spectrum disorders (ASDs; Haswell et al., 2009), attention deficit hyperactivity disorder (Piek and Pitcher, 1999), PTSD (Adrien et al., 2024; Rabellino et al., 2018; Payne et al., 2015; Linson et al., 2020), and depression (Buyukdura and McClintock, 2011; Cornell and Suarez, 1984). Note also that the motor feedforward mechanism is precisely the system that becomes dysfunctional in ASDs (Mosconi et al., 2015). Allowing for active engagement via multisensory interactions, GS may be implemented to increase the sense of self, sense of presence, BA and SA, and connectedness with the environment and with others (Ciaunica et al., 2021b; Ogden et al., 2006), for instance in patients with dissociation (e.g., depersonalization or derealization), a condition frequently found in patients with PTSD that makes them feel detached from their self, body, and the world (Sierra and Berrios, 1997; Ciaunica et al., 2021a). We suggest that GS-based multisensory and dynamic interactions with the physical and social environments may offer patients with dissociation a powerful tool to reconnect with their estranged and ‘detached’ selves, and to retrieve thereby the lost feeling of immersiveness, making patients feel more present in their bodies, and less solipsistically ‘trapped’ in their minds (Ciaunica et al., 2021a). This would help restoring the SO, BA and the SA that are altered in PTSD (Adrien et al., 2024).

Some limitations to this study should be noted. First, the sample size is smaller than ideal and a larger-sized replication would be needed to confirm these exploratory results. Additionally, we only used two classes of sounds (nature and machines). It would be important to replicate the study using a wider diversity of soundscapes. In this work, we permitted participants to improvise their movements to avoid artificial constraints; future studies may seek to control for specific movement patterns to study the effect of GS on BA, immersion, and embodiment for specific motor patterns (e.g., classical reflex arcs). Future studies should incorporate validated scales and tasks measuring BA (Mehling, 2020; Jones et al., 2020) to position these findings within the extant literature.

The small sample size not only restricts statistical power but also hinders our ability to detect more nuanced effects across different expertise levels. While the participants spanned novice to expert dancers, a larger, more heterogeneous sample would provide a better understanding of how GS affects body awareness (BA) and sense of agency (SA) across a wider population, potentially revealing subtle variations within and between expertise groups. The sample size also limits our ability to account for the full range of inter-individual variability, particularly in how participants engage with and respond to the sonification technology. Replication with a larger, more diverse sample is crucial to confirm these exploratory findings and improve the generalizability of the results.

Additionally, while self-reported measures provided valuable insight into subjective experiences, their reliance introduces potential biases. Including more objective physiological measures, such as galvanic skin response or movement accuracy tracking, could mitigate these biases and provide complementary data to support the subjective reports. Finally, expanding the range of soundscapes beyond the two categories (nature and machines) used in this study would allow us to explore whether different auditory modalities differentially affect BA and SA. These additions would deepen our understanding of the interaction between sound, movement, and self-perception, and further validate GS as a tool for mental health applications.

We therefore emphasize that this is an exploratory study and that the results presented here are preliminary, opening up research on the mental health potential of GS. Research has linked group dance and movement therapy with beneficial changes in psychiatric conditions, including a shift from self-awareness to awareness of others that leads to improved social interaction (Erfer and Ziv, 2006). A further iteration of the present study (and technology) adapted to a group setting could investigate finer-grained transitions in the process of increasing awareness of self and others through GS. In addition, future studies could compare differences between visual, auditory, and audiovisual feedback in BA. One area this could contribute to is dance education research, where it has been shown that adverse mental health and wellbeing outcomes can result from learning body movements in front of a mirror (i.e., visual reinforcement of proprioceptive feedback; Radell et al., 2020).

## 5 Conclusion

In this study, we explored the effect of GS on reports of well-being, BA, the SA and emotions in dance sessions. We found that across various levels of experience and throughout various types of sounds, GS reliably increased BA and made the experience more pleasurable than mere dancing and improvising. We discussed the role of proprioception, BA and the SA across multiple psychiatric conditions, especially in PTSD, and believe that replicating these results in psychiatric settings could have a high potential.

## Data Availability

The raw data supporting the conclusions of this article will be made available by the authors, without undue reservation.
